# Experimental Investigation and Prediction on Pressure Drop during Flow Boiling in Horizontal Microchannels

**DOI:** 10.3390/mi12050510

**Published:** 2021-05-01

**Authors:** Yan Huang, Bifen Shu, Shengnan Zhou, Qi Shi

**Affiliations:** Guangdong Provincial Key Laboratory of Photovoltaic Technology, School of Physics, Sun Yat-sen University, Guangzhou 510006, China; huangy235@mail2.sysu.edu.cn (Y.H.); zhoushn@mail2.sysu.edu.cn (S.Z.); shiq5@mail2.sysu.edu.cn (Q.S.)

**Keywords:** microchannel, two-phase flow, pressure drop, prediction, superficial gas flux

## Abstract

In this paper, two-phase pressure drop data were obtained for boiling in horizontal rectangular microchannels with a hydraulic diameter of 0.55 mm for R-134a over mass velocities from 790 to 1122 kg/(m^2^·s), heat fluxes from 0 to 31.08 kW/m^2^ and vapor qualities from 0 to 0.25. The experimental results show that the Chisholm parameter in the separated flow model relies heavily on the vapor quality, especially in the low vapor quality region (from 0 to 0.1), where the two-phase flow pattern is mainly bubbly and slug flow. Then, the measured pressure drop data are compared with those from six separated flow models. Based on the comparison result, the superficial gas flux *j_g_* is introduced in this paper to consider the comprehensive influence of mass velocity and vapor quality on two-phase flow pressure drop, and a new equation for the Chisholm parameter in the separated flow model is proposed as a function of the superficial gas flux *j_g_*. The mean absolute error (MAE) of the new flow correlation is 16.82%, which is significantly lower than the other correlations. Moreover, the applicability of the new expression has been verified by the experimental data in other literatures.

## 1. Introduction

With many cutting-edge technologies developing, the demand for compact heat exchangers that can dissipate enormous heat from small surface areas is growing [1,2,3]. Due to their compact structure and high heat exchange efficiency, microchannel heat exchangers are widely used in the fields of microelectronic mechanical systems (MEMS), aerospace and large-scale integrated circuit cooling [4,5]. In microchannel heat exchangers, boiling flow is considered to be a better choice than single-phase flow considering its high heat transfer efficiency and small wall temperature rises. Compared with conventional macroscopic channels heat exchangers, microchannel heat exchangers have a higher heat transfer coefficient [6,7]. However, researches show that the heat transfer and flow characteristics in a microchannel were different from those in a microchannel [8,9]. Hence, there is still a lot of work to be done to get a comprehensive understanding of heat transfer mechanism and flow characteristics in a microchannel device.

Generally, the frictional pressure drop increases in microchannels, and researches show that the characteristics of flow boiling pressure drop in microchannels are affected by a lot of factors. Tong et al. [10] studied the flow boiling pressure drop of four different diameters of tubes. They found that pressure drop increases with increasing mass velocity and heat flux. Huo et al. [11] also came to the same conclusion. While Park et al. [12] disagreed with this view, they pointed out that heat flux has little effect on pressure drop during flow boiling. Yam et al. [13] researched the effects of heat flux, mass velocity and saturation temperature on boiling pressure drop of R-134a in tubes with hydraulic diameter of 2 mm. They found that the measured pressure drop is higher as the mass velocity and the heat flux increased; they also mentioned that the effect of the changing saturated temperature on the pressure drop can be significant in a high vapor quality region (x > 0.65). Quan et al. [14] found that the two-phase frictional pressure drop in microchannels is influenced greatly by the hydraulic diameter, mass velocity and vapor quality. The pressure drop decreased as the hydraulic diameter increased, while increased with the increasing mass velocity and vapor quality. Balasubramanian et al. [15] also found that boiling pressure drop increases with increase in heat flux, and they attributed it to stronger wall friction and body drag effects. As the pressure drop increases in the boiling flow, the efficiency of the system decreases and the applicability of the device becomes limited. Therefore, the prediction of pressure drop is very important in the design of the two-phase flow system. For the study of pressure drop in two-phase flow, two main ways named the homogeneous model and separated flow models are referred. The homogeneous model is by far the simplest of pressure drop models and assumes that the two phases are mixed well and there is no slip between phases, the mixture behaves like a single-phase fluid with mean fluid properties depending on the vapor quality. Assuming thermodynamic equilibrium and uniform flow velocity and distribution, the mixture density can be given by:

(1) $$\frac{1}{\rho_{tp}}=\frac{x}{\rho_{g}}+\frac{1-x}{\rho_{l}}$$

The expressions used for the mixture viscosity are different in different homogenous models. Based on the assumptions of both uniform and equal phase velocities, the homogeneous flow model is expected to be suitable in bubbly and mist flow, where the slip velocity between the phases is small.

The separated flow models are fundamentally different from the homogeneous flow model, and it is assumed that the liquid phase and the vapor gas are separated and flow at different velocities. Most published works for predicting pressure drop in microchannels employ the separated flow models based on the Lockhart and Martinelli model [16]; in the model, the two-phase pressure drop can be obtained from:

(2) $${\left(-\frac{dP}{dz}\right)}_{tp}={\left(-\frac{dP}{dz}\right)}_{l}+C{\left[{\left(-\frac{dP}{dz}\right)}_{l}{\left(-\frac{dP}{dz}\right)}_{g}\right]}^{\frac{1}{2}}+{\left(-\frac{dP}{dz}\right)}_{g}$$

where *C* is the Chisholm parameter, being a measure of the interaction between the liquid and the gas. Equation (2) can be rewritten based on a two-phase multiplier as:

(3) $$\phi_{l}^{2}=1+\frac{C}{X}+\frac{1}{X^{2}}$$

where Martinelli parameter *X* is defined as:

(4) $$X^{2}={\left(\frac{dP}{dz}\right)}_{l}/{\left(\frac{dP}{dz}\right)}_{g}$$

and two-phase multiplier can be calculated by:

(5) $$\phi_{l}^{2}={\left(\frac{dP}{dz}\right)}_{tp}/{\left(\frac{dP}{dz}\right)}_{l}$$

Most studies used the separated flow model in order to research the two-phase flow more appropriately. Early separated flow model is suitable for predicting the frictional pressure drop of two-phase flow in conventional macroscopic channels, but in the later research process, it is found that the two-phase flow in microchannels exhibits drastically different flow behaviors from its counterpart in conventional macroscopic channels. According to studies by Sadatomi et al. [17], Lockhart and Martinelli’s [16] correlation can be used to calculate the frictional pressure drop of two-phase flow, for conventional size channels, *C* = 21, and for narrow channels, *C* = 0. In order to make Lockhart and Martinelli’s [16] correlation more suitable for the microchannel pressure drop calculation, the channel size, physical parameters, the vapor quality, the mass velocity, etc., were taken into account in later research. Mishima and Hibiki [18] used air–water mixture as the working fluid to study the frictional pressure drop of the two-phase flow in aluminum and glass tubes with an inner diameter of 1–4 mm, and they found that the Chisholm parameter is related to the inner diameter of the tubes. Later researchers showed its high accuracy in mini/microchannels [19,20,21,22,23,24]. Qu and Mudawar [22] used water as the working fluid to study the frictional pressure drop of water phase change in 21 parallel microchannels with a channel size of 231 × 713 μm, and they found that C is related to the mass velocity. They also improved the pressure drop correlation based on Mishima and Hibiki’s [18]. Zhang et al. [25] collected a variety of data from the literature to evaluate previous correlations, and applied the neural network analysis method to propose a new correlation for the Chisholm parameter as a function of the Laplace parameter *La*. Lim et al. [26] performed experimental study with a hydraulic diameter of 0.5 mm, using water as the working fluid. They found that the two-phase multiplier decreases with the increase of mass velocity, and a new correlation model based on the Chisholm parameter was proposed as a function of the two-phase Reynolds and Weber numbers. Later, Choi et al. [27] carried out an experimental study on the boiling pressure drop using FC-72 as the working fluid in 15 parallel channels with a length of 60 mm and size of 0.45 mm × 0.2 mm. They found that the two-phase frictional multiplier largely depends on the vapor quality, and the frictional pressure drop increases as the vapor quality increases. Meanwhile, the two-phase frictional multiplier was modified with the dimensionless parameters such as Reynolds number, Weber number and Martinelli parameter, and a new correlation was proposed.

Other existing two-phase frictional pressure drop prediction correlations are essentially based on experiments and fitted with experimental data. Tran et al. [28] studied two-phase flow pressure drop of R-134a, R-12 and R-113 in square and round tubes with diameters of 2.46 mm and 2.92 mm under the pressure of 138 kPa to 856 kPa, and proposed that the pressure drop calculations, which is suitable for conventional size channels, is not suitable for their experimental condition. They also proposed a new correlation for pressure drop calculation. Zhang and Webb [29] used R-134a, R-22 and R-404a as the working fluid to study the two-phase flow pressure drop in aluminum tubes with the hydraulic diameter of 2.13 mm, and in copper tubes with hydraulic diameter of 6.25 mm and 3.25 mm. They pointed out that the Fridel’s [30] correlation cannot accurately predict the pressure drop of their experiments, and proposed a new correlation based on the Fridel’s [30]. At present, the accuracy and applicability of the proposed pressure drop prediction correlations are limited due to the different working materials and working conditions in different researches.

In the present study, based on the previous work of two-phase flow pattern and heat exchange [31], R-134a is used as the working fluid to conduct a boiling flow experiment in a rectangular channel with a hydraulic diameter of 0.55 mm. Two-phase pressure drop data were obtained for R-134a evaporation in horizontal rectangular microchannels (the hydraulic diameter D_h_ = 0.55 mm) with mass velocities from 790 to 1122 kg/(m^2^·s), heat fluxes from 0 to 31.08 kW/m^2^ and vapor qualities from 0 to 0.25. The Reynolds number of gas phase is from 11 to 7490 and the Reynolds number of liquid phase is from 1680 to 2915. These data have been compared against six two-phase frictional pressure drop prediction models. Based on the experimental data, the superficial gas flux $j_{g}$ is introduced in this paper to consider the comprehensive influence of mass velocity and vapor quality on two-phase flow pressure drop. The frictional pressure drop was reproduced well with a new equation for the Chisholm parameter in the separated flow model as a function of superficial gas flux $j_{g}$. The mean absolute error MAE of the new flow correlation is 16.82%, which is significantly lower than the other correlations.

## 2. Materials and Methods

### 2.1. Experimental Methods

**Figure 1:**
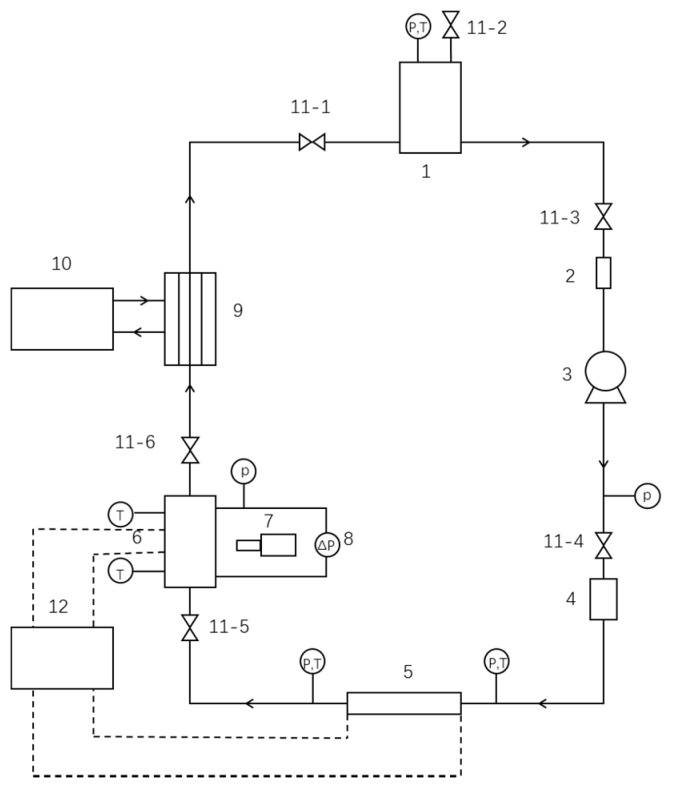
The schematic diagram of the experimental devices. 1, Reservoir; 2, filter; 3, micropump; 4, flowmeter; 5, pre-heater; 6, test-section; 7, camera; 8, differential pressure transmitter; 9, plate heat exchanger; 10, water cooling system; 11-1~11-6, valves; 12, AC power source.

The schematic of the two-phase experimental apparatus in the present study is shown in Figure 1. A two-phase flow loop is constructed, in which R-134a is used as the working fluid. A reservoir is employed to maintain the working fluid at a constant reference pressure and to separate the vapor and liquid. R-134a is circulated in the entire loop utilizing a magnetic micro gear pump. After leaving the reservoir, the fluid passes through filters, pump, rotameter, pre-heater, test section and condenser, and eventually returns to the reservoir. The refrigerant is preheated by adjusting the power of the preheater to change the inlet vapor quality, and then flows into the microchannel test section for heat exchange. Both the pre-heater and the test section are heated electrically, which ensures a uniform heat flux. The reservoir is placed at a temperature of 20 °C, and the pressure is adjusted to ensure that the working fluid of the reservoir outlet is at a saturated state. The entire experimental loop is wrapped with asbestos to reduce air convection heat transfer and radiant heat loss.

**Figure 2:**
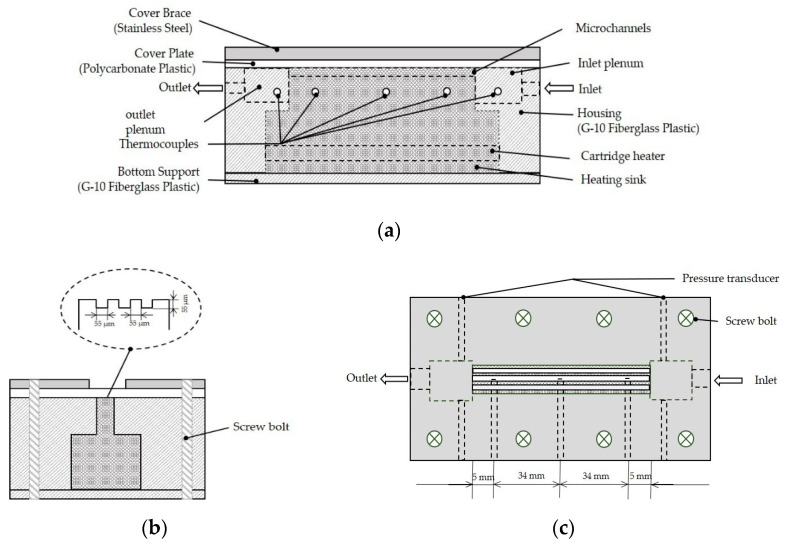
(**a**) Cross-sectional view of the test section; (**b**) construction of the test-section; and (**c**) top view.

In the test-section, copper is used as the heating substrate for the microchannels, and three rectangular microchannels with a hydraulic diameter of 0.55 mm are processed on the top. The channel parameters are shown in Table 1. Figure 2 shows the construction and details of the test-section.

Before the experiment, a vacuum pump was used to evacuate the experimental system. When the system pressure dropped below 0.1 kPa, the vacuum pump was turned off. To make sure the whole flow loop is checked for no leakage, the system must remain with the pressure unchanged for 12 h, and then the experiment was conducted. The inlet pressure of working fluid is measured by a pressure sensor, whose measurement uncertainty is less than 0.5%. The differential pressure of the test-section is measured by a differential pressure transmitter with a measurement uncertainty less than 0.6%. The measurement uncertainty of the volume flowmeter is about 5.5%. Two RTDs are installed in the inlet and outlet plenum to monitor the inlet and outlet fluid temperature, and three type-K thermocouples are inserted in the heat sink to monitor the heat sink wall temperature. Uncertainty associated with type-K thermocouples and RTDs are smaller than 0.5 °C and 0.3 °C, respectively. Table 2 shows the maximum uncertainty of the system.

### 2.2. Data Processing

The two-phase pressure drop is comprised of the frictional, the accelerational and gravitational components. In horizontal tubes, the gravitational component can be negligible.

(6) $$\Delta p_{tp}=\Delta p_{tp,fric}+\Delta p_{tp,a}$$

The accelerational component can be given by Equation (7) [18]

(7) $$\Delta p_{tp,a}=G^{2}\left\{\left[\frac{{\left(1-x_{out}\right)}^{2}}{\rho_{l}\left(1-\alpha_{out}\right)}+\frac{x_{out}^{2}}{\rho_{g}\alpha_{out}}\right]-\left[\frac{{\left(1-x_{in}\right)}^{2}}{\rho_{l}\left(1-\alpha_{in}\right)}+\frac{x_{in}^{2}}{\rho_{g}\alpha_{in}}\right]\right\}$$

The inlet and outlet vapor quality can be given by Equations (8) and (9)

(8) $$x_{in}=\frac{h_{lo}+\frac{\dot{P}}{\dot{m}}-h_{l,in}}{h_{lg,in}}$$

(9) $$x_{out}=x_{in}+\frac{\dot{Q}}{\dot{m}h_{lg,out}}$$

The void fraction can be obtained by Zivi’s [32] model

(10) $$\alpha =\frac{1}{1+\left(\frac{1-x}{x}\right){\left(\frac{\rho_{g}}{\rho_{l}}\right)}^{\frac{2}{3}}}$$

For the homogeneous model, the void fraction is related to the vapor quality by the relation

(11) $$\alpha ={\left[1+\left(\frac{1-x}{x}\right)\left(\frac{\rho_{g}}{\rho_{l}}\right)\right]}^{-1}$$

In Lockhart and Martinelli’ model, the void model can be obtained from

(12) $$\alpha ={\left[1+0.28{\left(\frac{1-x}{x}\right)}^{0.64}{\left(\frac{\rho_{g}}{\rho_{l}}\right)}^{0.36}{\left(\frac{\mu_{l}}{\mu_{g}}\right)}^{0.07}\right]}^{-1}$$

**Figure 3:**
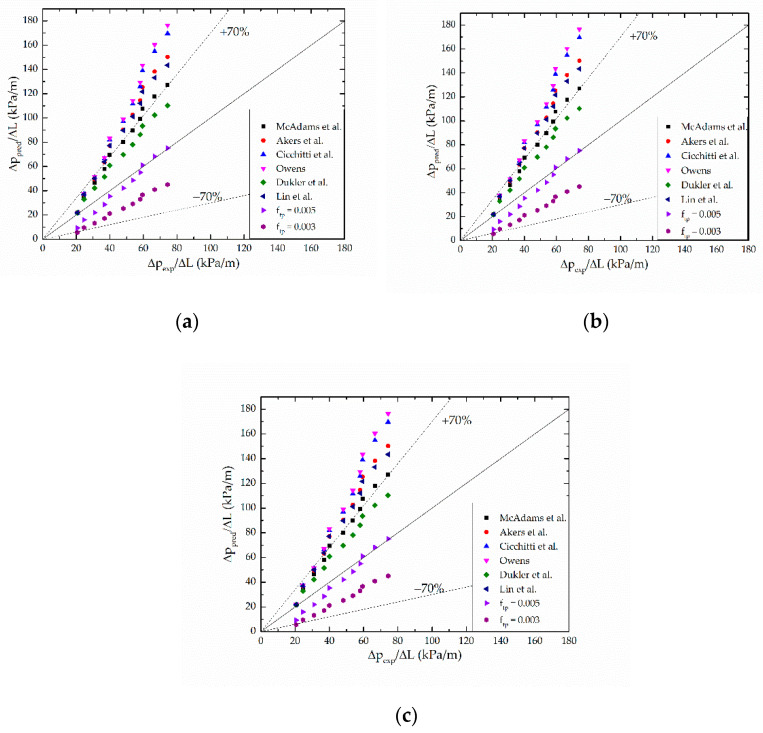
Comparison of predictions of homogeneous models with present experimental frictional pressure drop data calculated based on (**a**) Zivi’s model, (**b**) homogeneous model, and (**c**) Lockhart and Martinelli’ model.

In order to check the sensitivity of different void fraction models to frictional pressure drop, eight homogeneous models [33,34,35,36,37,38,39,40] are employed to compare Equation (10) to Equation (12). Figure 3 shows the comparison of the present frictional pressure drop data to the predictions based on these models. The frictional pressure drop data in Figure 3a are based on Zivi’s model for void fraction, and Figure 3b,c are based on the homogeneous model and Lockhart and Martinelli’s model. Figure 3 shows that the performance of all approaches is almost the same—the void fractions values calculated by the three models are almost the same, resulting in approximative frictional pressure drops—while the frictional pressure drop based on Zivi’s model is slightly closer to the predictions. Thus, the accelerational pressure drop will be calculated according to Zivi’s void fraction model.

When the working fluid flows into the test-section, the cross-section contracts, which causes increased flow velocity and increased pressure drop. When the working fluid flows out of the channels, the cross-section expands, which causes decreased flow velocity and pressure drop. Therefore, the experimentally measured microchannel pressure drop can be obtained from:

(13) $$\Delta p_{tot}=\Delta p_{c}+\Delta p_{tp}-\Delta p_{e}$$

The contraction pressure loss and expansion recovery at the inlet and the outlet of the microchannels are determined from relations by Collier and Thome [41]:

(14) $$\Delta p_{c}=\frac{G^{2}}{2\rho_{l}}\left(\frac{\rho_{l}x_{in}}{\rho_{tp}}+1\right)\left[{\left(\frac{1}{C_{c}}-1\right)}^{2}+\left(1-\sigma_{c}^{2}\right)\right]$$

and

(15) $$\Delta p_{e}=G^{2}\left(\frac{x_{out}}{\rho_{tp}}+\frac{1}{\rho_{l}}\right)\sigma_{e}\left(1-\sigma_{e}\right)$$

where the mixture density $\rho_{tp}$ and the contraction coefficient $C_{c}$ can be given by Equations (1) and (16) [42]

(16) $$C_{c}=1-\frac{1-\sigma_{c}}{2.08\left(1-\sigma_{c}\right)+0.5371}$$

To sum up, the frictional pressure drop can be obtained from:

(17) $$\Delta p_{tp,fric}=\Delta p_{tot}-\Delta p_{c}+\Delta p_{e}-\Delta p_{tp,a}$$

For two-phase frictional pressure drop, Lockhart and Martinelli [16] gave a correlation based on a two-phase multiplier for the liquid phase, which is defined as:

(18) $$\phi_{l}^{2}={\left(\frac{dP}{dz}\right)}_{tp,fric}/{\left(\frac{dP}{dz}\right)}_{l}$$

the two-phase multiplier $\phi_{l}^{2}$ and the Martinelli parameter X can be obtained from Equation (3) to Equation (5).

The frictional pressure drop gradient for all liquid flow and all gas flow can be obtained from:

(19) $${\left(\frac{dP}{dz}\right)}_{l}=\frac{2f_{l}{\left(1-x\right)}^{2}G^{2}}{D_{h}\rho_{l}}$$

(20) $${\left(\frac{dP}{dz}\right)}_{g}=\frac{2f_{g}x^{2}G^{2}}{D_{h}\rho_{g}}$$

where the friction factors can be given by Equation (21) to Equation (23).

For laminar flows, the friction factor is given as a function of aspect ratio by Shah and London [43]

(21) $$fRe=24\left(1-1.3553\beta +1.9467\beta^{2}-1.7012\beta^{3}+0.9564\beta^{4}-0.2537\beta^{5}\right),\;Re<2000$$

For turbulent flows, the expression of Blasius is used:

(22) $$f=0.079Re^{-0.25},\;2000<Re<20,000$$

(23) $$f=0.046Re^{-0.2},\;Re\geq 20,000$$

## 3. Results

### 3.1. Pressure Drop Results

The experiment was conducted under the condition over mass velocities from 790 to 1122 kg/(m^2^·s), heat fluxes from 0 to 31.08 kW/m^2^ and vapor qualities from 0 to 0.25, and 263 effective experimental data points were obtained.

**Figure 4:**
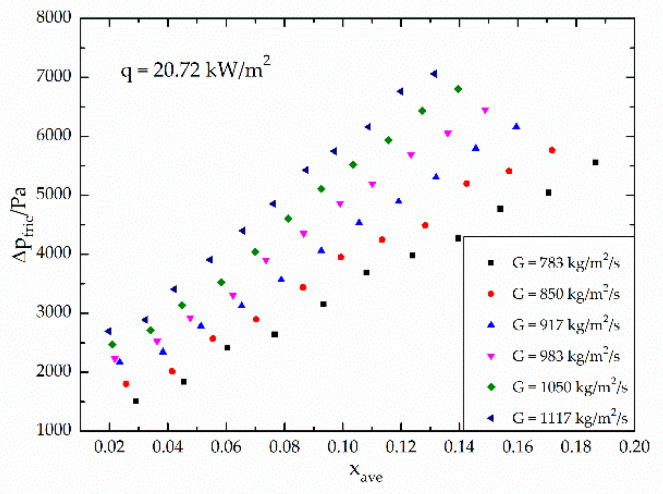
Variation of frictional pressure drop with respect to average vapor quality under different mass velocities.

**Figure 5:**
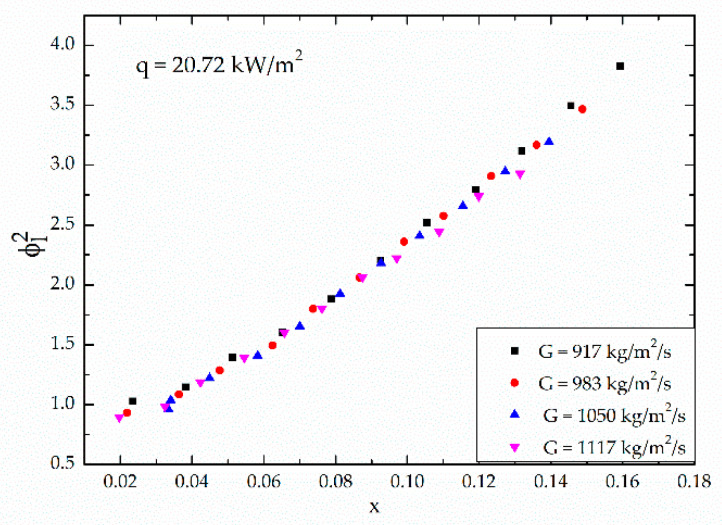
Variation of the two-phase frictional multiplier ($\phi_{l}^{2}$) with respect to the vapor quality under different mass velocities.

**Figure 6:**
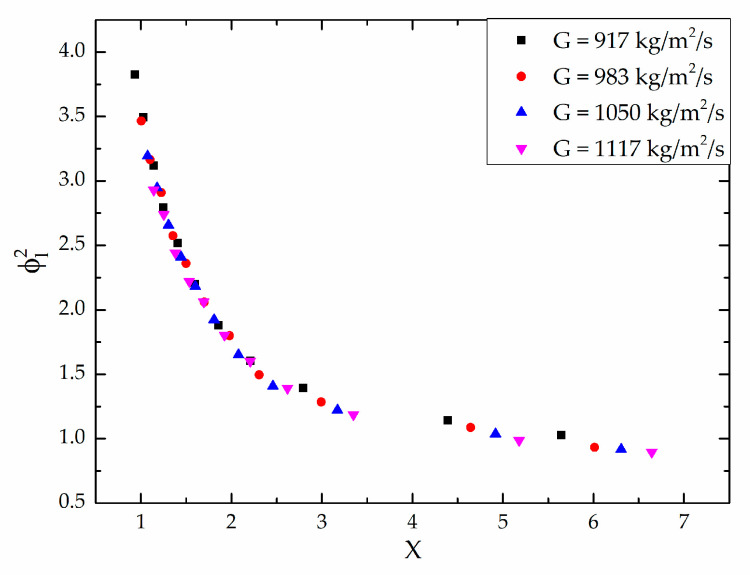
Variation of the two-phase frictional multiplier ($\phi_{l}^{2}$) with respect to the Lockhart and Martinelli parameter under different mass velocities.

Figure 4 shows the variation of the frictional pressure drop with respect to the average vapor quality. The average vapor quality is defined as the average value of inlet and outlet vapor quality. As shown in Figure 4, the frictional pressure drop is influenced by both the vapor quality and the mass velocity. As the vapor quality and the mass velocity increases, the frictional pressure drop increases. Figure 5 shows the variation of the two-phase frictional multiplier ($\phi_{l}^{2}$) with respect to the vapor quality in the middle of the channel. It indicates that the two-phase frictional multiplier is related to the vapor quality. As the vapor quality increases, the two-phase frictional multiplier increases almost linearly, which quite agrees with Choi et al. [27]. However, the mass velocity has little effect on the two-phase frictional multiplier. Figure 6 shows the deviation of the two-phase frictional multiplier ($\phi_{l}^{2}$) between Lockhart and Martinelli’s [16] correlation and experimental data, versus the Martinelli parameter, X. It also shows the mass velocity has little effect on the two-phase frictional multiplier.

### 3.2. Comparison with Different Pressure Drop Correlations

**Figure 7:**
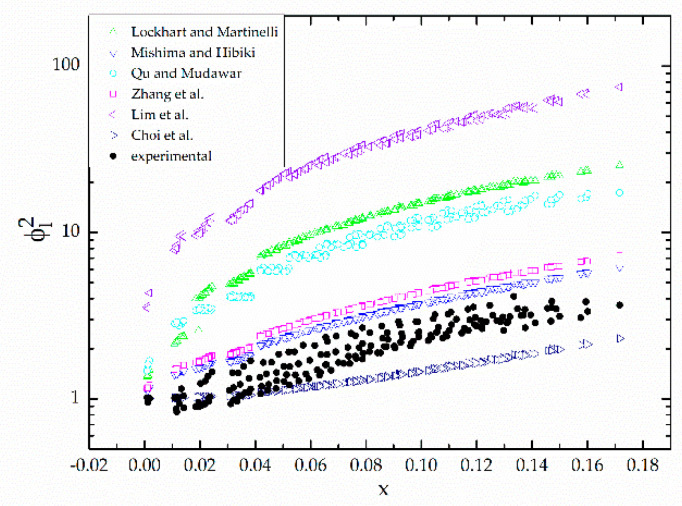
Comparison between the measured two-phase multiplier and previous correlations.

The comparison of experimental and predicted values of the two-phase frictional multiplier varied with the vapor quality is shown in Figure 7. The predicted values are calculated from six various correlations. These correlations are all obtained by modifying the correlation of Lockhart and Martinelli [16]. Table 3 provides details of the correlations as well as their accuracy in predicting the present data. The accuracy of individual correlations above is measured by mean absolute error, which is defined as:

(24) $$\mathrm{MAE}=\frac{1}{N}\sum \frac{\left|\Delta P_{pred}-\Delta P_{exp}\right|}{\Delta P_{exp}}\times 100\%$$

**Figure 8:**
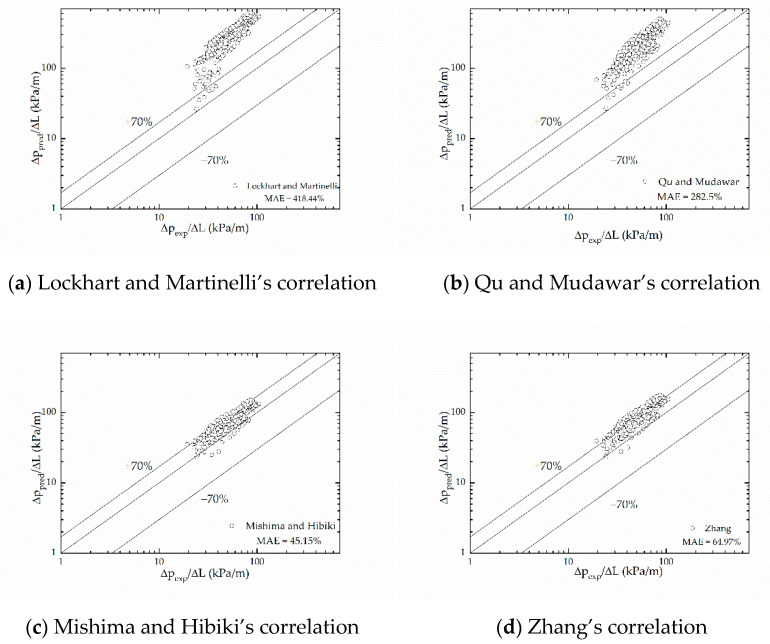

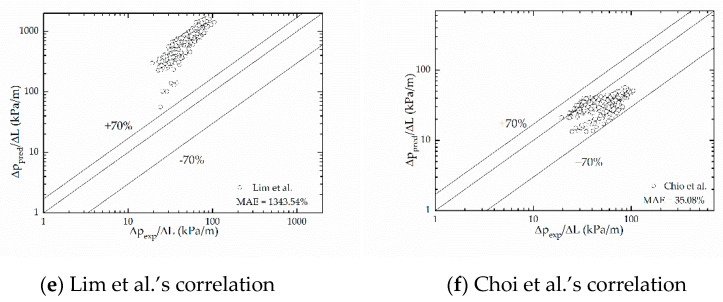
Comparison of measured frictional pressure drop data with predictions of separated flow correlations proposed by (**a**) Lockhart and Martinelli, (**b**) Qu and Mudawar, (**c**) Mishima and Hibiki, (**d**) Zhang, (**e**) Lim et al. and (**f**) Choi et al.

Figure 8 compares the present pressure drop data to predictions based on six aforementioned correlations. Figure 7 and Figure 8 both show that most correlations overpredicted the two-phase frictional multiplier compared with the experimental results, while the correlation of Choi et al. [27] underpredicted slightly. Among these correlations, the correlation of Choi et al. [27] predicted most accurately. Mishima and Hibiki’s [18] correlation that modified the Lockhart and Martinelli’s [16] correlation based on the hydraulic diameter of the tubes also performs fairly well, as well as Zhang’s [25] correlation, which is based on the dimensionless number *La* parameter.

Lockhart and Martinelli’s [16] correlation is widely used in conventional size tubes. However, it predicts worst in the present study. It may be attributed to the existence of size effect, surface effect, and wall effect, etc. in microchannels. For this reason, Lockhart and Martinelli’s [16] correlation is no longer applicable for microchannels. Figure 7 depicts a smaller C in the present study than that in Lockhart and Martinelli’s correlation, which agrees with Sadamoti [17], while diverges from Lim et al.’s opinion [26]. The possible reason is that Lim et al. used water, oil, etc. as the working fluid, and substances like those have a higher viscosity than R-134a, which is used as the working fluid in the present study. As the viscosity increases, the flow resistance gets larger, which results in a larger C. In addition, the surface tension of substances like water is also higher, so it has a poor wettability, and bubbles generated in fluids like water need more time to escape from the wall surface, which causes an increasing flow resistance.

Mishima and Hibiki’s [18] correlation, Qu and Mudawar’s [22] correlation and Zhang’s [25] correlation share similar modality, while Mishima and Hibiki’s [18] correlation based on the hydraulic diameter of the tubes predicts best, with a mean absolute error 45.15%. Zhang’s [25] correlation based on parameter *La* follows, with a mean absolute error 64.97%. Qu and Mudawar’s [22] correlation considering the impact of the hydraulic diameter and the mass velocity has a larger mean absolute error, which is 282.5%, and it might be reasonable to conclude that the effect of mass velocity on the frictional pressure drop is exaggerated (as Figure 4 and Figure 5 show, the mass velocity has little effect on the two-phase frictional multiplier). Zhang’s [25] correlation, although doesn’t predict best, covers more experimental conditions, so it has a wider applicability. Zhang combined the experimental data of many scholars with the hydraulic diameter from 0.007 mm to 6.25 mm, and many kinds of working fluid like water, water/air, R-22, R-134a, etc. The neuron analysis method was used and the dimensionless parameter *La* was taken into consideration to study the effect of surface tension. Compared to macrochannels, the effect of surface tension increases in microchannels [44,45] and it is important to take the surface tension into consideration, so Zhang’s correlation has a great referential significance.

Lim et al. [26] and Choi et al. [27] both presumed the Chisholm parameter to be a function of dimensionless numbers, Reynolds number and Weber number. Lim et al. chose dimensionless numbers based on the mixed physical properties of liquid-vapor of the homogeneous flow model, while Choi et al. chose those based on liquid properties. Lim et al.’s [26] correlation gives a large mean absolute, which may due to the higher viscosity and the surface tension of water, and the size effect further magnifies the difference. Choi et al.’s [27] correlation shows a relatively good prediction for present pressure drop data with a mean absolute error 35.08%; the working fluid used is closer to R134a in properties, and the correlation considered the influence of inertial force, viscous force and surface tension.

## 4. New Correlation

### 4.1. Influence of Local Vapor Quality on the Chisholm Parameter

**Figure 9:**
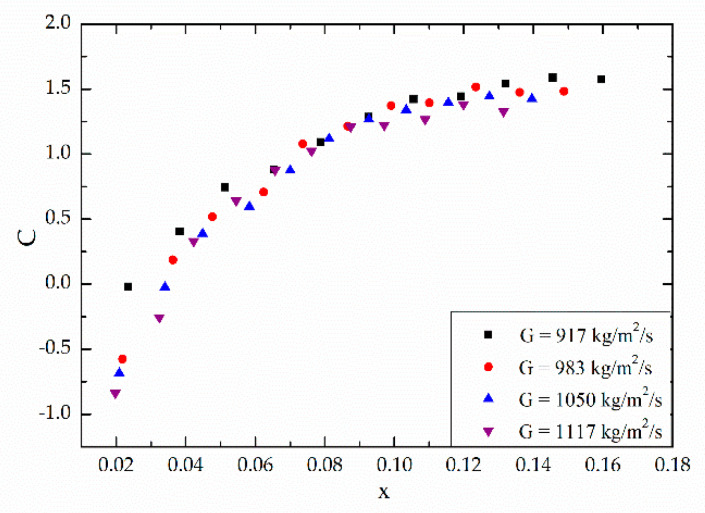
Variation of C under different mass velocities, versus local vapor quality, x, for q = 20.72 kW/m^2^.

It is found in our experimental study that the vapor quality has major effect on the pressure drop and two-phase multiplier, shown in Figure 4 and Figure 5, and the mass velocity has little effect on C, while the vapor quality’s effect is obvious, shown in Figure 9. It also shows that when the vapor quality x < 0.1, the Chisholm parameter increases with increasing vapor quality, and when the vapor quality x > 0.1, the Chisholm parameter tends to be stable. Referring to our previous work [31], there is a relationship between the quality and the flow pattern. When x < 0.1, the flow pattern in the channel is mainly bubbly flow and slug flow. At first, the gas occupies a small volume and bubbles fusion rarely occurs. After the formation, the bubbles are washed out of the channel quickly and the flow resistance is small. As the vapor quality increases, slug flow occurs, the gas occupies more space, restricting bubbles to form, which results in an increasing flow resistance. When x > 0.1, vapor in the channel start to merge and form churn flow and not-fully-developed annular flow, where gas occupies a large volume and liquid forms a film on the wall surface. The flow resistance occurs on the wall surface, so the frictional pressure drop change with the vapor quality is not so obvious.

### 4.2. Improved Correlation

A new approach was developed to improve the prediction accuracy of pressure drop in two-phase microchannels in low vapor quality region. Since the flow patterns are various and chaotic—from bubbly flow to not-fully-developed annular flow, the separated flow model seems to be more appropriate than the homogeneous model in this study. Considering the experimental results and the mechanism of two-phase flow in microchannel, the superficial gas flux is introduced to improve the pressure drop prediction correlation.

(25) $$j_{g}=\frac{Gx}{\rho_{g}}$$

By introducing the superficial gas flux, the influence of the mass velocity and the vapor quality are considered comprehensively.

**Figure 10:**
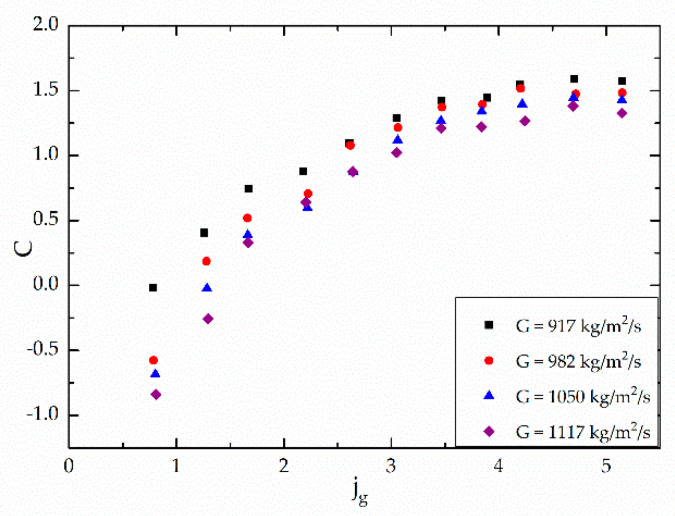
Variation of C under different mass velocities, versus superficial gas flux, $j_{g}$, for q = 20.72 kW/m^2^.

Figure 10 shows the variation of C under different mass velocities, versus superficial gas flux with the heat flux of 20.72 kW/m^2^. As shown in Figure 10, the Chisholm parameter is obviously affected by the superficial gas flux.

Among the aforementioned separated models, Zhang’s [25] correlation has a similar form with the other two models—Mishima and Hibiki’s [18] and Qu and Mudawar’s [22]. However, the correlation of Zhang [25] considers the surface tension and covers more data points of experimental conditions, which has great reference significance. In this study, the Chisholm parameter is defined as a function to consider the influence of the inertial force, surface tension, vapor quality and mass velocity based on Zhang’s [25] correlation:

(26) $$C_{modify}=21\left[1-\exp\left(-\frac{0.358}{La}\right)\right]\left(aj_{g}+b\right)$$

**Figure 11:**
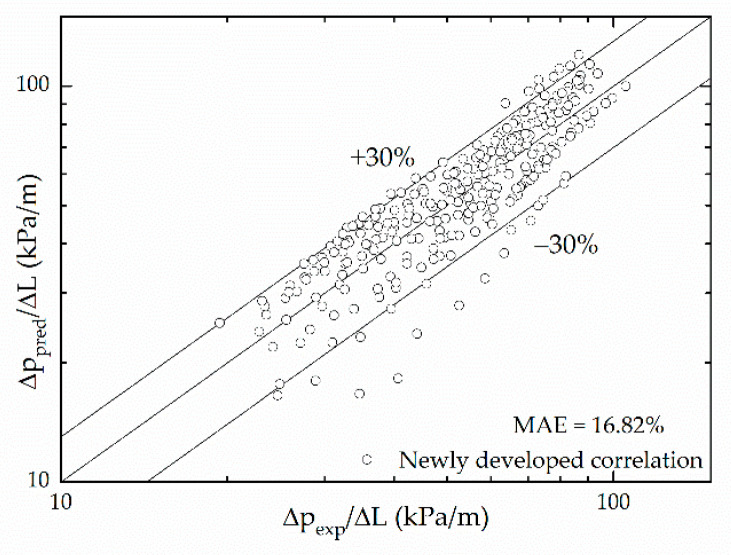
Comparison of pressure frictional pressure drop data with predictions of the separated flow correlation with the new C.

Fitting the 263 experimental data points—over mass velocities from 790 to 1122 kg/(m^2^·s), heat fluxes from 0 to 31.08 kW/m^2^ and vapor qualities from 0 to 0.25—to the equation, a = 0.06548 and b = 0.17033. Figure 11 compares the present pressure drop to predictions based on Lockhart and Martinelli’s correlation with the new Chisholm parameter. It shows that the accuracy of the correlation prediction is significantly improved, and most data points are within 30% deviation, with the mean absolute error MAE = 16.82%.

**Figure 12:**
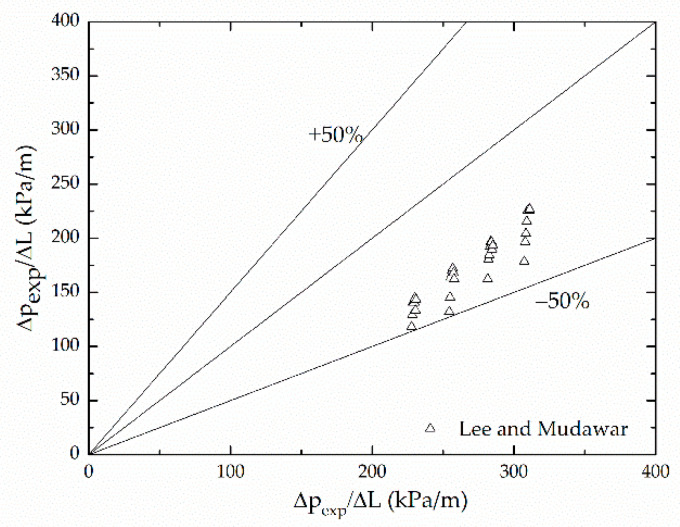
Comparison of new correlation with Lee and Mudawar’s microchannel R-134a data.

To enhance the predictive capability of the new correlation, R-134a data of Lee and Mudawar’s [19] were examined. Figure 12 shows the present correlation is very effective at predicting the microchannel R-134a of Lee and Mudawar.

## 5. Conclusions

In this study, R-134a is used as the experimental working fluid, and a flow boiling experiment is conducted in a horizontal rectangular with a hydraulic diameter of 0.55 mm. Six existing correlations are evaluated and a new correlation is proposed. Key findings from this study are as follows:

1. Among six separated flow models, most correlations overpredicted the frictional pressure drop compared with the experimental data of flow boiling in horizontal microchannels in this paper, while the correlation of Choi et al. underpredicted slightly. Correlations proposed by Mishima and Hibiki, based on the hydraulic diameter, and Zhang, related to the Laplace parameter *La*, share similar form and work comparatively well in predicting pressure drop of two-phase within an acceptable mean absolute error. Zhang’s correlation, although doesn’t predict best, covers more experimental conditions, so it has a wider applicability. However, Zhang’s correlation only took the effect of surface tension into consideration, regardless of the operating conditions. To consider the effect of operating conditions, a new correlation is proposed based on Zhang’s correlation in this paper.
2. The vapor quality is found to have a significant influence on the Chisholm parameter in the separated flow model, and this may be due to its influence on the flow pattern. When x < 0.1, the flow pattern is mainly bubbly flow and slug flow, the Chisholm parameter increases with the increasing vapor quality. When x > 0.1, the bubbles merge to form churn flow and not-fully-developed annular flow, and the Chisholm parameter remains nearly unchanged.
3. The superficial gas flux $j_{g}$ is introduced to consider the comprehensive influence of mass velocity and vapor quality on two-phase flow pressure drop, and a new equation for the Chisholm parameter in the separated flow model is proposed as a function of the superficial gas flux $j_{g}$. The mean absolute error MAE of the new flow correlation is 16.82%, which is significantly lower than the other correlations. Moreover, the applicability of the new expression has been verified by the experimental data in other literatures.

## Figures and Tables

**Table 1:** The channel parameters.

Number of Channels	Channel Width, *W_ch_*/mm	Channel Length, *L*/mm	Channel Depth, *H_ch_*/mm
3	0.55	78	0.55

**Table 2:** Measurement error and uncertainty.

Parameter	Maximum Uncertainty
Pressure	0.5%
Differential pressure	0.6%
Fluid temperature	0.3 °C
Wall temperature	0.5 °C
Heat flux	0.5%
Mass velocity	5.7%
Vapor quality	7.2%

**Table 3:** Two-phase frictional pressure gradient correlations based on the separated flow model and corresponding MAE in predicting present frictional pressure drop data.

Author(s)	Equation	Remarks	MAE
Lockhart and Martinelli [16]	${\left(\frac{dp}{dz}\right)}_{tp,fric}=\phi_{l}^{2}{\left(\frac{dp}{dz}\right)}_{l}$ ${\left(\frac{dp}{dz}\right)}_{l}=\frac{2f_{l}{\left(1-x\right)}^{2}G^{2}}{D_{h}\rho_{l}}$ $\phi_{l}^{2}=1+\frac{C}{X}+\frac{1}{X^{2}}$ $X^{2}={\left(\frac{dp}{dz}\right)}_{l}/{\left(\frac{dp}{dz}\right)}_{g}$ $C_{vv}=5$ $C_{tv}=10$ $C_{vt}=12$ $C_{tt}=20$	*D_h_* = 1.49–25.83 mm adiabatic fluid; water, oils, hydrocarbons; round tubes	418.44%
Mishima and Hibiki [18]	Using the Lockhart and Martinelli correlation $C_{M\&H}=21\left[1-\exp\left(-0.319D_{h}\right)\right];D_{h}\left[mm\right]$	*D_h_* = 1.05–4.08 mm adiabatic fluid: air/water; round tube	45.15%
Qu and Mudawar [22]	Using the Lockhart and Martinelli correlation $C_{Q\&M}=21\left[1-\exp\left(-0.319D_{h}\right)\right]\left(0.00418G+0.0613\right)$	*D_h_* = 0.087 mm flow boiling multi-channels fluids; water; rectangular tubes	282.5%
Zhang [25]	Using the Lockhart and Martinelli correlation $C_{Zhang}=21\left[1-\exp\left(-0.358/La\right)\right]$ $La=\sqrt{\frac{\sigma }{g\left(\rho_{l}-\rho_{g}\right)D_{h}^{2}}}$	*D_h_* = 0.007–6.25 mm adiabatic/diabatic fluids; water, water/air, R-22, R-134a, etc.; round/rectangular tubes	64.97%
Lim et al. [26]	Using the Lockhart and Martinelli correlation $C_{Lim}=0.71Re_{tp}^{0.91}We_{tp}^{-0.655}$	*D_h_* = 0.5 mm flow boiling fluid; water; rectangular tube	1343.54%
Choi et al. [27]	Using the Lockhart and Martinelli correlation $C_{Chio}=0.05Re_{lo}^{0.68}We_{lo}^{-0.34}X^{-1.32}$	0.45 mm × 0.2 mm flow boiling multi-channels fluids; FC-72; rectangular tubes	35.08%

## Data Availability

The data presented in this study are available on request from the corresponding author. The data are not publicly available due to privacy.

## Nomenclature

f	friction factor [-]	La	the Laplace number [-]
h	enthalpy [J/kg]		$La=\sqrt{\frac{\sigma }{g\left(\rho_{l}-\rho_{g}\right)D_{h}^{2}}}$
$h_{lg}$	latent heat of vaporization [J/kg]		
$j_{g}$	superficial gas flux [m/s]	MAE	mean absolute error
$\dot{m}$	mass flow rate [kg/s]	N	number of experimental data points
p	pressure [Pa]	$\dot{P}$	heating power of the preheating section [W]
v	flow velocity [m/s]		
x	thermodynamic equilibrium vapor quality [-]	$\dot{Q}$	heating power of the test-section [W]
		Re	the Reynolds number [-]
z	coordinate along microchannel [mm]	$W_{ch}$	channel width [mm]
C	the Chisholm parameter [-]	We	the Weber number [-]
$C_{c}$	contraction coefficient [-]		$We=\frac{\rho v^{2}D_{h}}{\sigma }$
$D_{h}$	hydraulic diameter [mm]		
G	mass velocity [kg/(m^2^·s)]	X	the Martinelli parameter [-]
$H_{ch}$	channel depth [mm]		$X^{2}={\left(\frac{dP}{dz}\right)}_{l}/{\left(\frac{dP}{dz}\right)}_{g}$
L	channel length [mm]		
Greek Symbols			
α	void fraction [-]	$\sigma_{e}$	expansion area ratio [-]
β	channel aspect ratio [-]	Δ	difference [-]
ρ	density [kg/m^3^]	ϕ	two-phase pressure drop
$\rho_{tp}$	mixture density [kg/m^3^]		multiplier [-]
$\sigma_{c}$	contraction area ratio [-]		
Subscripts			
a	accelerational	lo	liquid only
ave	average	out	microchannel outlet
c	contraction	pre	predicted
e	expansion	tot	total
exp	experimental	tp	two-phase
fric	frictional	tt	turbulent liquid-turbulent vapor
g	saturated vapor	tv	turbulent liquid-laminar vapor
in	microchannel inlet	vt	laminar liquid-turbulent vapor
l	saturated liquid	vv	laminar liquid-laminar vapor
